# The nitric oxide paradox: antimicrobial and inhibitor of antibiotic efficacy

**DOI:** 10.1042/ETLS20230114

**Published:** 2023-11-17

**Authors:** Calum M. Webster, Mark Shepherd

**Affiliations:** School of Biosciences, RAPID Group, University of Kent, Canterbury CT2 7NJ, U.K.

**Keywords:** antibiotics, bioenergetics, nitric oxide, respiration

## Abstract

It is well-known that antibiotics target energy-consuming processes and a significant body of research now supports the conclusion that the metabolic state of bacteria can have a profound impact upon the efficacy of antibiotics. Several articles implicate bacterial energetics and the respiratory inhibitor nitric oxide (NO) in this process, although pinpointing the precise mechanism for how NO can diminish the potency of a range of antibiotics through modulating bacterial energy metabolism has proved challenging. Herein, we introduce the role of NO during infection, consider known links between NO and antibiotic efficacy, and discuss potential mechanisms via which NO present at the site of infection could mediate these effects through controlling bacterial energetics. This perspective article highlights an important relationship between NO and antibiotic action that has largely been overlooked and outlines future considerations for the development of new drugs and therapies that target bacterial energy metabolism.

## Introduction

Many studies have reported bacterial metabolism to elicit a profound impact upon susceptibility to antibiotics and there is now a widely accepted link between the two [1]. However, while several articles have reported the modulation of antibiotic efficacy by NO, we are yet to piece together the details of a generalised mechanism for how NO exposure can diminish the potency of numerous antibiotic classes. Herein, we aim to assemble a narrative on this and consider the broader implications of these findings for future drug discovery.

## NO is an antimicrobial respiratory inhibitor

Before one can consider the influence of NO upon antibiotic efficacy, it is important to understand the roles for NO during infection. NO is a diatomic free radical that is produced by the host immune system in response to bacterial infection. Exposure to NO and its congeners is both bactericidal and bacteriostatic, the effects being mediated through a range of well-known mechanisms, including modification of iron sulphur clusters [2], nitrosylation of thiol groups [3], nitration of tyrosine residues in the presence of superoxide [4], and through the inhibition of aerobic respiration via binding to haem cofactors of oxygen-dependent terminal oxidoreductases [5]. Bacteria can produce a variety of responses to NO [6], including systems involved in NO-detoxification, iron-sulphur (Fe-S) cluster repair, and the synthesis of NO-tolerant respiratory complexes such as cytochrome *bd*. While the toxic effects of NO and bacterial responses are wide ranging, this section focusses on the interplay between NO found at the site of infection and systems involved in bacterial energetics.

**Figure 1.**
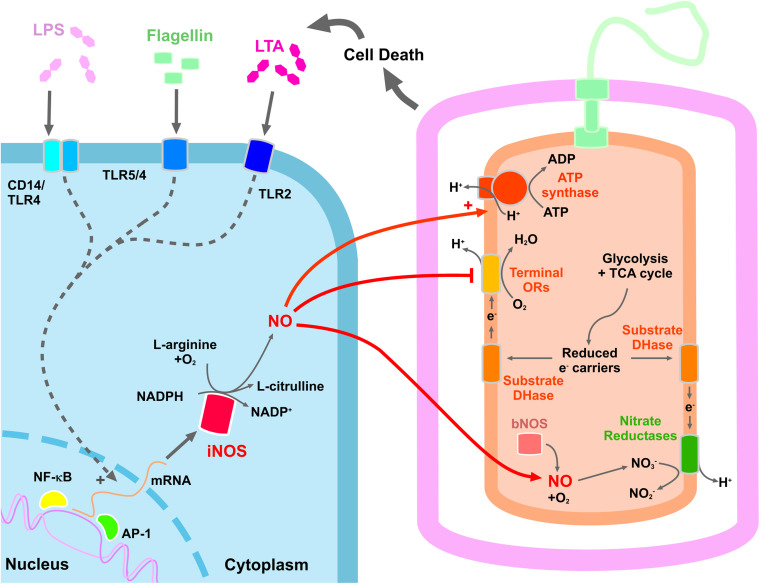
Interplay between NO and bacterial energetics during infection. Key mechanisms in bacterial-mediated induction of host iNOS production in immune cells are shown, including the bacterial PAMPs lipopolysaccharide (LPS), flagellin and lipoteichoic acid (LTA) that are released during bacterial cell death and activate TLR-mediated signalling pathways. This causes activated NF-κB or AP-1 to enter the nucleus and up-regulate the transcription of NOS2 encoding the iNOS enzyme, which catalyses the conversion of l-arginine to l-citrulline and NO. NO can diffuse across the host and bacterial membranes bind to haem cofactors on oxygen-dependent oxidoreductases that shuts down aerobic respiration. NO is also produced by some bacteria via endogenous bacterial NOS enzymes. Auto-oxidation of NO may result in the production of nitrate that can be used as a respiratory electron acceptor. During NO exposure, the ATP synthase may also contribute to the proton-motive force by operating in reverse. Abbreviations: AP-1, activator protein-1; DHase, dehydrogenase; iNOS, inducible nitric oxide synthase; NF-κB, nuclear factor kappa B; ORs, oxidoreductases. N.B. not all mechanisms will operate in all bacteria.

Figure 1 depicts the interaction between a ‘generic’ invading bacterial pathogen and a host immune cell. The host immune response results in death of some of the bacterial cells, which releases a variety of immunogenic ‘pathogen-associated molecular patterns’ (PAMPs), including lipopolysaccharide (LPS) from the cell envelope [7], flagellin protein from the flagellum [8] or lipoteichoic acid (LTA) from the cell wall of Gram-positives [9]. This in turn can activate signalling cascades involving Toll-Like Receptors (TLRs), the precise details of which are reviewed elsewhere and are beyond the scope of this article [10–12]. In short, this signalling cascade stimulates the transcription of the NOS2 gene encoding an inducible nitric oxide synthase (iNOS), leading to the production of NO from l-arginine. This NO can then diffuse out of the immune cell and into the bacterium to inhibit oxygen-dependent terminal oxidoreductases. While these haem-binding respiratory complexes are generally very sensitive to NO, other systems involved in bacterial energetics have been shown to combat nitrosative stress. For example, when *Staphylococcus aureus* is exposed to nitrosative stress the ATP synthase operates in reverse to maintain membrane potential [13], and a similar mechanism has also been reported for *Salmonella* pathogenesis [14]. In addition, Gram-positive pathogens such as *Bacillus anthracis* and *S. aureus* produce NO endogenously using a bacterial nitric oxide synthase (bNOS) enzyme, which is important during pathogenesis [15,16]. In *S. aureus*, endogenously produced NO inhibits aerobic respiration while promoting the utilisation of the TCA cycle to produce NADH, which can then be used for reduction in nitrate by the respiratory chain (N.B. in the presence of oxygen, NO is auto-oxidised to nitrate). Similarly, *E. coli* and *Salmonella* have been shown to exploit iNOS-derived nitrate as a respiratory electron acceptor, which accelerates growth and helps these Gram-negatives to out-compete commensal gut bacteria [17,18]. All of these potential mechanisms are depicted in Figure 1.

## Bacterial energetics, NO, and the efficacy of bactericidal antibiotics

While the relationship between NO and bacterial growth and survival during infection is already complicated, another layer of complexity exists when one considers the substantial evidence that supports the influence of bacterial metabolic activity and energetics upon susceptibility to antibiotics [1,19]. Indeed, Stokes et al. [1] draw from a broad collection of literature to assemble a clear narrative on how bactericidal antibiotics can enhance metabolic activity, which in turn can itself contribute to cell death. Hence, as NO is a well-known respiratory inhibitor and can dramatically influence bacterial metabolism and energetics, it is likely that NO can modulate antibiotic efficacy through modulating these bacterial processes. This notion is explored via consideration of two postulates:

1. The efficacy of certain antibiotics is diminished by NO.
2. Antibiotics elicit elevated metabolism/bioenergetics as part of their bactericidal mechanism, which can be counteracted by the presence of NO.

To assess the first of the two postulates one can consider early work in this area that used phenotype microarrays to demonstrate that endogenous NO production via the *B. subtilis* bNOS enzyme alleviated antibiotic-mediated growth inhibition caused by a wide range of antibiotic classes, including β-lactams, quinolones and aminoglycosides [20]. However, only acriflavine was shown to be directly modified by NO to a less toxic product, and the hypothesised role for NO in alleviating oxidative stress that may be induced by β-lactams, quinolones and aminoglycosides was not unequivocally resolved in this study [20]. That said, subsequent work has confirmed that NO can diminish the efficacy of aminoglycosides [21–23], β-lactams [20,24], and quinolones [20], which provides compelling evidence to support acceptance of postulate 1. However, there is a notable exception to this trend where NO enhances the lethality of antibiotics in bacterial biofilms [25–27]. Although NO is a well-known signalling molecule involved in biofilm dispersal [28–32], the potentiation of antibiotic efficacy is potentially related to ease of antibiotic access and an increase in metabolic activity during a return to planktonic growth. Indeed, NO has been shown to up-regulate cellular phosphodiesterases via interaction with cell receptors leading to increased c-di-GMP degradation, which in turn produces the metabolic changes required for biofilm dispersal [29]. Hence, improved antibiotic diffusion [27] and metabolic changes [33] have been cited as reasons for the enhanced lethality of antibiotics when used in conjunction with NO in biofilms.

The second postulate may be considered by reviewing the influence of aminoglycosides, β-lactams and quinolones upon the energetic state of the bacterial cell. All three classes of antibiotic have been shown to elevate respiratory oxygen consumption [34] and to elicit the formation of reactive oxygen species (ROS) that may contribute to cell death [35–38]. Conversely, previous studies conclude that the majority of superoxide and peroxide are derived from the autooxidation of non-respiratory flavoproteins [39–41], although given the scale of conflicting evidence in the literature it would seem premature to exclude the respiratory chain as a source of ROS. A good example of antibiotic-mediated increases in metabolic activity is the β-lactam mecillinam, which has been shown to induce an energetically expensive futile cycle that contributes to cell death [42]. Inducing respiratory metabolism has also been shown to enhance the efficacy of quinolones against *Mycobacterium smegmatis* and metabolically repressed *E. coli* and *S. aureus* [43]. However, other studies with *E. coli* have reported that β-lactams, quinolones and aminoglycosides do not elevate peroxide production nor promote accumulation of intracellular free iron, and lethality persists in the absence of oxygen [44]. This supports the hypothesis that mechanisms of lethality for these antibiotic classes are independent of ROS. Indeed, for aminoglycosides alternative mechanisms of lethality have been reported, including membrane hyperpolarisation via reversal of ATP synthase activity [45–47] and through elevated aminoglycoside uptake that is dependent upon Fe-S cluster biosynthesis [48]. The electrical component of the PMF has been shown to be related to aminoglycoside uptake in *Bacillus subtilis*, *S. aureus, E. coli and Salmonella* [49,50], and the role of PMF in aminoglycoside lethality is supported by recent work using membrane uncouplers and demonstration that gentamicin does not impose significant oxidative stress upon *E. coli* [45]. Also, voltage-gated calcium influxes have been used to demonstrate aminoglycoside-mediated increases in membrane polarity, and membrane polarisation was shown to be unnecessary for aminoglycoside uptake but essential for bactericidal effects [47].

**Figure 2.**
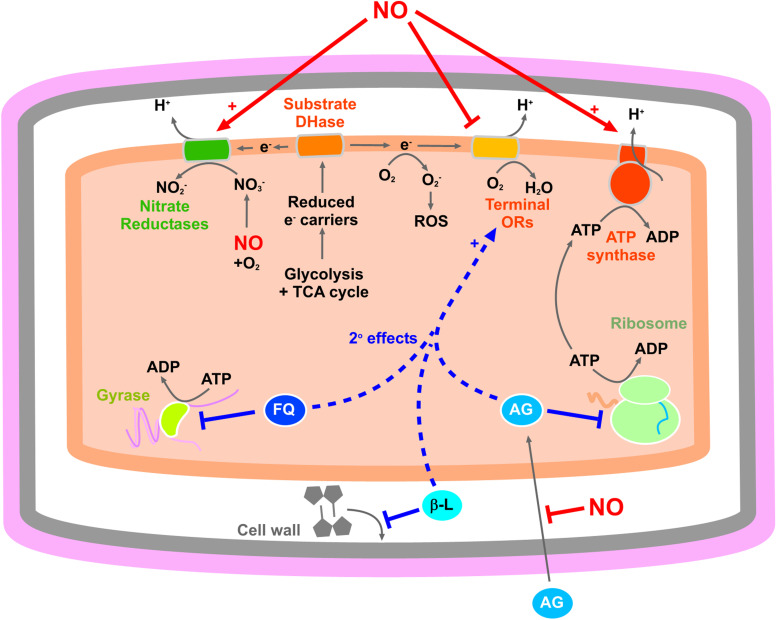
NO diminishes antibiotic efficacy potentially via modulating energetics. Known mechanisms of how NO modulates bacterial energetics are shown (from Figure 1) alongside their potential impact upon the secondary effects of bactericidal antibiotics that cause cell death. Exposure to fluoroquinolones (FQ), β-lactam (β-L) and aminoglycosides all have the common secondary effect of elevating aerobic respiration and ROS generation, which in turn contributes to cell death. Aminoglycosides may also cause ATP accumulation, which can drive the ATP synthase in reverse and elicit cell death via membrane hyperpolarisation. Also, NO-mediated perturbations in energy metabolism can decrease aminoglycoside uptake. Proton translocation across the membrane is via a vectorial mechanism linked to quinol oxidation for nitrate reductases, whereas some oxygen-dependent complexes can also pump protons. Abbreviations: DHase, dehydrogenase; ORs, oxidoreductases; ROS, reactive oxygen species.

Figure 2 depicts potential routes for how NO may interfere with energetic processes associated with cell death for these three antibiotic classes. NO could diminish ROS generation through direct inhibition of aerobic respiration. In addition, NO-mediated inhibition of aerobic respiratory complexes might also diminish the PMF and help to counteract the excessive PMF that is generated during aminoglycoside exposure [47], and lowering the PMF may also lead to reduced aminoglycoside uptake [22]. However, it is more difficult to envisage how NO-mediated stimulation of nitrate reductase activity [17,18] might alleviate aminoglycoside-mediated membrane hyperpolarisation’, as one might expect that these electrogenic respiratory complexes would exacerbate proton efflux. However, this process of course depends upon the oxygen levels, as nitrate reductases are maximally expressed under anaerobic conditions in the presence of nitrate, but most studies on NO and aminoglycosides have been performed under aerobic conditions where nitrate reductases are poorly expressed.

Thus far, we have mainly focussed on the interaction between NO, aerobic respiration and antimicrobial susceptibility, which excludes our knowledge of antibiotics killing bacteria under anaerobic conditions. There is a large literature that addresses the need for alternative antibiotic regimens to treat anaerobes [51,52] but there has been very little focus on the influence of NO upon antibiotic efficacy during anaerobiosis, except for a recent study reporting that an NO donor can diminish the potency of gentamicin under anoxic growth [23]. Given the ability of some bacteria to both produce and consume NO during anaerobic respiration using nitrite as an electron donor [53–55], and bearing in mind the well-known anaerobic NO-resistance machineries in bacteria [56], there appears to be an important gap in our knowledge that relates to how NO may more broadly impact upon antibiotic treatments during anaerobiosis. One might assume that oxygen-dependent terminal oxidoreductases and ROS would not play a significant role in antibiotic-mediated bacterial killing during anaerobiosis, but an unequivocal elucidation of anaerobic bactericidal mechanisms remains elusive.

## Conclusions

While targeting bacterial energetics is a very promising route for the development of new antimicrobials [57], careful considerations would need to be made before using these in conjunction with antibiotic classes that require a working respiratory chain for optimal efficacy. There is also a huge literature on NO releasers as novel antimicrobials [58–60], although little consideration has been given to how NO therapies might impact upon conventional antibiotic treatments, or indeed how iNOS-derived NO might affect antibiotic action at the site of infection. Hence, in light of the dramatic effects that NO has upon antibiotic efficacy, it would be sensible to give greater consideration to how NO and bacterial energetics can influence the efficacy of new antibiotics.

## Open Access

Open access for this article was enabled by the participation of University of Kent in an all-inclusive *Read & Publish* agreement with Portland Press and the Biochemical Society under a transformative agreement with JISC.

## Abbreviations

Fe-S: iron-sulphur
LPS: lipopolysaccharide
LTA: lipoteichoic acid
NO: nitric oxide
PAMP: pathogen-associated molecular pattern
PMF: proton-motive force
ROS: reactive oxygen species
TLR: Toll-like receptor
